# Supplementary figures and images for: Ubiquitin activation is essential for schizont maturation in Plasmodium falciparum blood-stage development
Source: PLoS Pathog. 2020 Jun 22;16(6):e1008640. doi: 10.1371/journal.ppat.1008640 (PMC7332102; doi:10.1371/journal.ppat.1008640)

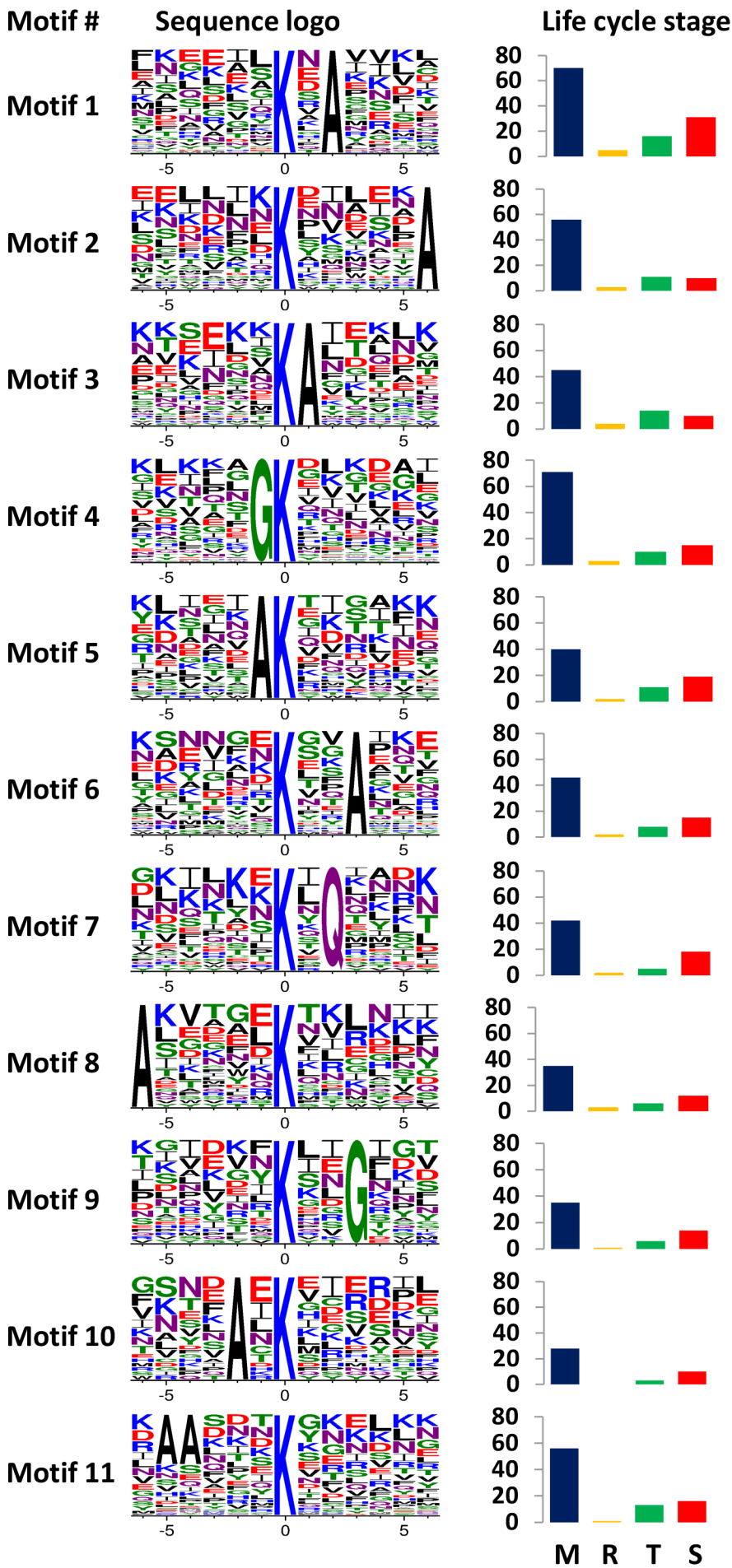

Supplement: S1 Fig — Each motif (obtained from S1 Dataset) consists of 13 amino acids with the ubiquitylated lysine at the centre. The size of the amino acid one-letter code represents the abundance of that residue in the sequence at that position. On the right side of each motif its abundance detected in merozoite (M, blue), ring (R, yellow), trophozoite (T, green) and schizont (S, red) stages is shown. (PDF) [file ppat.1008640.s001.pdf]

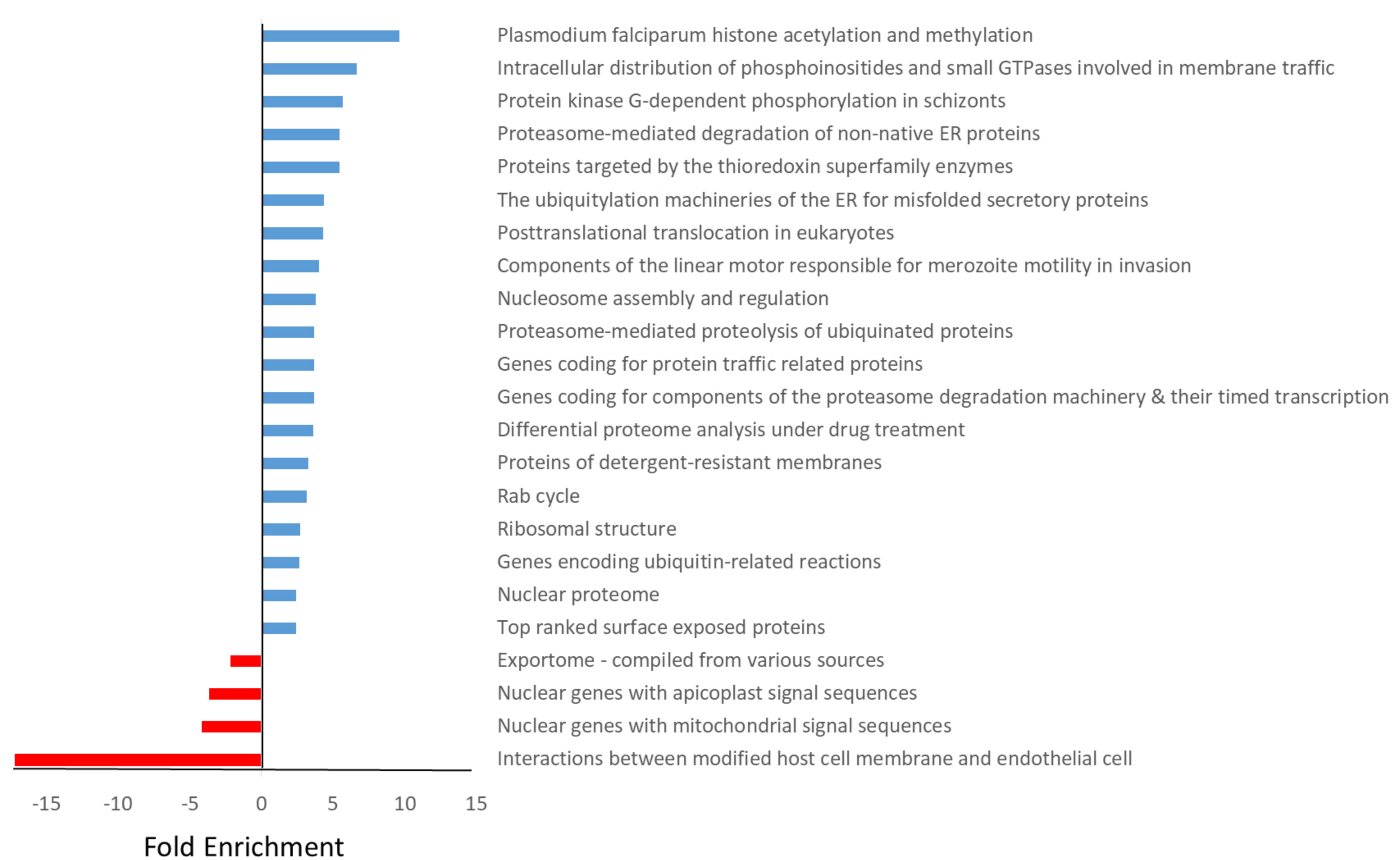

Supplement: S2 Fig — Significantly enriched pathways are in blue and downregulated pathways are in red, with the fold-enrichment indicated on the x-axis. (PDF) [file ppat.1008640.s002.pdf]

E1

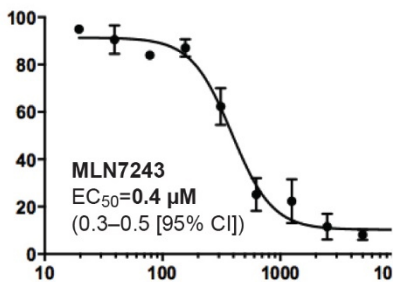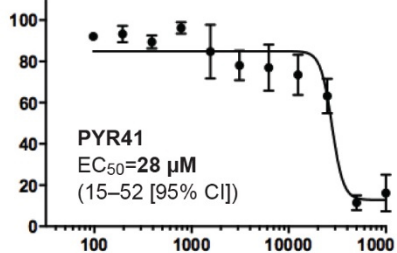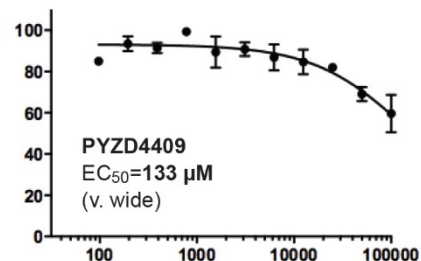

E2

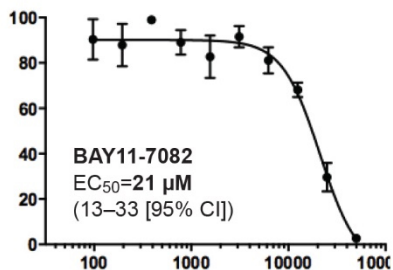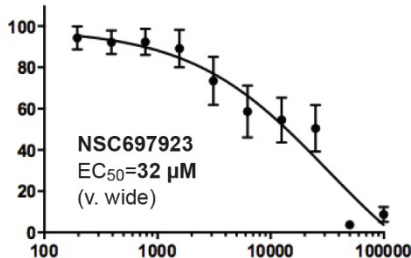

E3

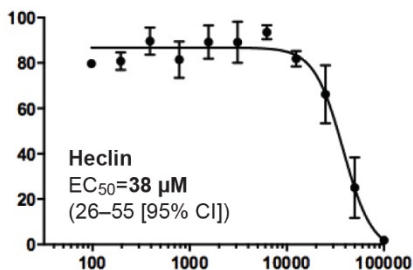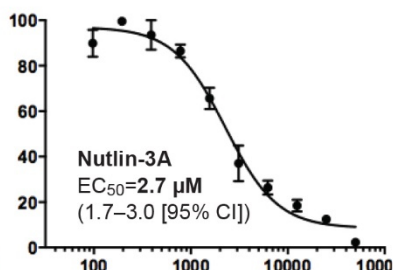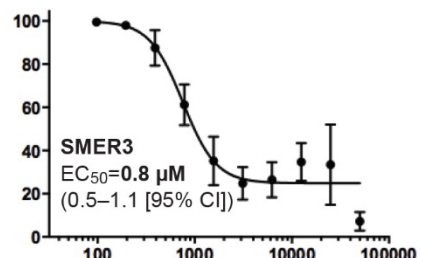

DUB

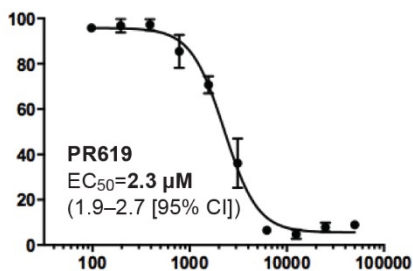

Inhibitor concentration [nM]

Supplement: S3 Fig — Cultures of synchronised parasites were grown in the presence of compounds for two cycles and then parasite growth was measured using a SYBR Green fluorescence-based plate assay. For each inhibitor an EC50 was calculated from the curve and is presented in Table 1. The results shown are means of triplicate samples from three independent experiments (+/- standard deviation). (PDF) [file ppat.1008640.s003.pdf]

a.

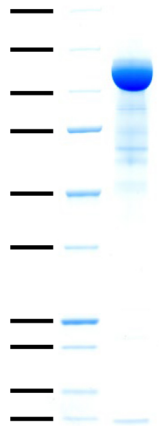

b.

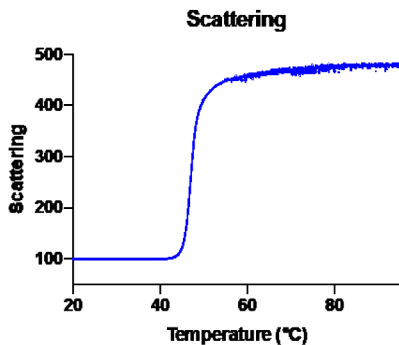

c.

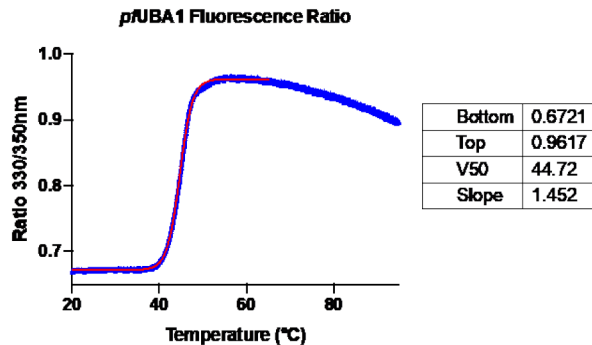

d.

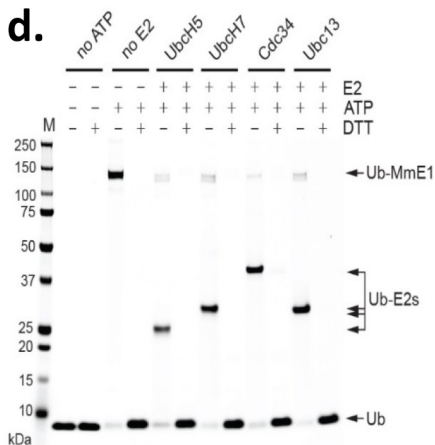

e.

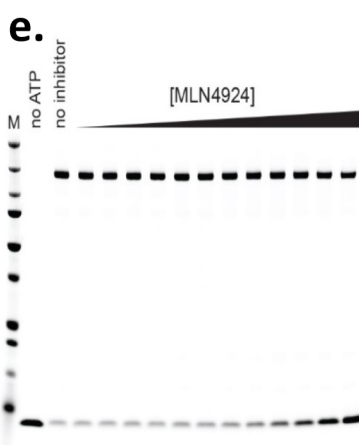

f.

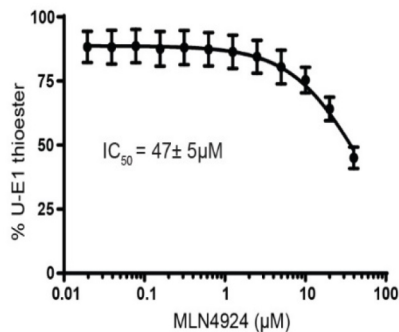

Supplement: S5 Fig — (a) Purification and stability of recombinant PfUBA1. The protein was expressed in the baculovirus-insect cell system and purified on Talon and MonoQ resins; analysis of final product by Coomassie blue stained SDS-PAGE. The thermal stability of the protein was analysed by (b) light scattering and (c) fluorescence to identify the melting temperature. (d) Validation of the in vitro thioesterification and transthioesterification assays: Mus musculus UBA1 is thioesterified by fluorescently labelled ubiquitin in the presence of ATP, and transthioesterifies various human E2s. (e) the lack of activity of a Nedd8 E1 inhibitor against PfUBA1; MLN4924, added in doubling dilutions, is a poor inhibitor of PfUBA1 thioesterification. (f) The data from panel (e) enabled the IC50 of MLN4924 to be calculated. Data are shown as mean ± standard deviation from technical triplicates. (PDF) [file ppat.1008640.s005.pdf]
