# Supplementary material for: Ubiquitin activation is essential for schizont maturation in Plasmodium falciparum blood-stage development
Source: PLoS Pathog. 2020 Jun 22;16(6):e1008640. doi: 10.1371/journal.ppat.1008640 (PMC7332102; doi:10.1371/journal.ppat.1008640)
Supplement: S4 Fig — The amino acid coding sequence was back-translated to nucleic acid sequence using codons preferentially used in Escherichia coli, and a synthetic gene was produced and cloned into a plasmid vector. (PDF) [file ppat.1008640.s004.pdf]

T E N K I D T D L Y S R Q L G T Y G F D L M N K L V K L N I L I I N V K G V G L E C A K N L I L S G P
1 GGATCCAACCGAGAACAAAATTGATACCGATCTGTATAGCCGTCAGCTGGGCACCTATGGTTTTGATCTGATGAATAAACTGGTGAAACTGAACATCCTGATCATCAATGTTAAAGGTGTTGGTCTGGAATGCGCCAAAAATCTGATTCTGAGCGGTCCG

Q S V C I Y D N D I C D I S D I G V N F Y I N E K D V E D K S C R S D A V L K E L Q E L N N Y V H I Y N Y
161 CAGAGCGTTTGTATTTATGATAATGATATTTGCGACATCAGCGACATCGGCGTGAACTTTTATATCAACGAAAAAGACGTGGAAGATAAGAGCTGTCGTAGTGATGCAGTTCTGAAAGAACTGCAAGAACTGAACAACTACGTGCACATCTATAACTATA

K G T I E K N W L E N F D V V I C C D I N K E D L I K Y N N M I R G I D K K R I A F L S C N I Y G L C G Y I
321 AAGGCACCATCGAAAAGAACTGGCTGGAAAATTTTGATGTGGTGATTTGCTGCGATATCAACAAAGAGGATCTGATCAAGTATAACAACATGATCCGTGGCATCGACAAAAAACGTATTGCATTTCTGAGCTGCAACATTTATGGTCTGTGCGGTTATAT

F V D F N K E F I C Y D S N G E Q V K S C N V S K I S K E L E G K V S F D F D K T S P F E E G D Y V Q F S
481 CTTCGTGGACTTCAATAAAGAGTTCATCTGCTACGATAGCAATGGCGAACAGGTTAAAAGCTGCAATGTTAGCAAAATCAGCAAAGAGCTGGAAGGCAAAGTCAGCTTTGATTTTGATAAAACGAGCCCGTTTGAAGAGGGTGATTATGTGCAGTTTAGC

N V E G M T E I N N K I Y K I K N L K K Y T F E I G D T S L Y S E Y I K G G I C T Q V K K H L K L N F Y P
641 AATGTTGAAGGCATGACCGAAATCAACAACAAAATCTATAAAATCAAAAACCTGAAAAAGTACACCTTCGAGATTGGTGATACCAGCCTGTATAGCGAATATATCAAAGGTGGTATTTGCACCCAGGTGAAAAAACATCTGAAGCTGAATTTCTATCCGT

Y E Y I C V N P L N N E N I S N N E Q K H N Q N D N H F L D T C N N I I Y E N I P Q P N S F I I S D Y A K F
801 ACGAGTATATTTGCGTGAATCCGCTGAATAACGAAAACATCAGCAACAACGAACAGAAGCATAACCAGAACGATAACCATTTTCTGGATACCTGCAACAACATCATCTATGAAAACATTCCGCAGCCGAACAGCTTTATCATTAGCGATTATGCCAAATT

D M S N H L H Y S I Q A L K W Y E L Q N E K G L P E N S D E D A L E K I Y N Y A V T L N N K D K E E K K S
961 TGATATGAGCAACCATCTGCACTATAGCATTCAGGCACTGAAATGGTATGAACTGCAGAATGAAAAAGGTCTGCCGGAAAATAGTGATGAAGATGCCCTGGAAAAAATCTACAATTATGCAGTGACCCTGAACAACAAGGACAAAGAAGAGAAAAAAAGC

Y A V E Q L K K D V V Y N V C R Y S K S H I A P V A S F F G G L L A Q E V I K F T G K Y M P I Y Q L L Y L
1121 TACGCAGTGGAACAGCTGAAAAAAGATGTTGTGTATAATGTGTGCCGCTACAGCAAAAGCCATATTGCACCGGTTGCAAGCTTTTTTGGTGGTCTGCTGGCACAAGAAGTGATCAAATTTACCGGCAAATATATGCCGATTTACCAGCTGCTGTATCTGG

D F F E C I S L N E K V D I N E I K K M N C K N D N I I T V F G K S F Q K K L N N L N V F L V G S G A L G C
1281 ATTTTTTTGAATGCATTAGCCTGAACGAGAAGGTGGATATTAACGAGATCAAAAAGATGAATTGCAAAAACGATAACATCATCACCGTGTTCGGTAAAAGCTTCCAGAAAAAACTGAATAACCTGAACGTGTTTCTGGTTGGTAGCGGTGCACTGGGTTG

E Y A K L F S L L D M C T R N S E Q N T N L N Q N N I D N N L A C C G K L T I T D N D N I E V S N L N R Q
1441 TGAATATGCAAAACTGTTTAGCCTGCTGGATATGTGTACCCGTAATAGCGAACAGAATACCAATCTGAACCAGAACAACATCGATAACAATCTGGCATGTTGTGGCAAACTGACCATTACCGATAATGACAATATTGAGGTGAGCAATCTGAATCGCCAG

F L F R R E H V G K S K S L V S S E I I K K K N N N M H V Q S L E T K V G A E N E H I F N E E F W T K Q N
1601 TTTCTGTTTCGTCGTGAACATGTGGGTAAAAGCAAAAGTCTGGTTAGCAGCGAGATCATCAAAAAAAAGAACAACAACATGCACGTCCAGAGCCTGGAAACCAAAGTTGGTGCAGAAAATGAACACATCTTCAACGAAGAATTCTGGACCAAACAGAACA

I I V N A L D N I Q A R Q Y V D N K C V W Y S K P L F E S G T L G T K G N V Q V I I P Y L T Q S Y N D S Y D
1761 TTATTGTGAACGCCCTGGATAATATTCAAGCCCGTCAGTATGTTGATAACAAATGCGTTTGGTATAGCAAACCGCTGTTTGAAAGCGGCACCCTGGGCACCAAAGGTAATGTTCAGGTGATTATTCCGTATCTGACCCAGAGCTATAATGATAGCTATGA

P P E D S I P L C T L K H F P Y D I V H T I E Y A R D I F Q G L F Y N T P L S I K Q F L N D K E E Y I N K
1921 CCCTCCGGAAGATAGCATTCCGCTGTGTACCCTGAAACATTTTCCGTATGATATTGTGCACACCATTGAATATGCCCGTGATATTTTTCAGGGCCTGTTTTATAACACACCGCTGAGCATCAAACAGTTTCTGAACGATAAAGAAGAGTACATCAACAAG

I Q E E G N N A S L L E N L Q N V I N S L K E I S S Q C N F D F C I K K S V E L F H N N F I N Q I N Q L L
2081 ATTCAAGAAGAAGGTAATAACGCCAGTCTGCTGGAAAACCTGCAGAACGTGATTAATAGCCTGAAAGAAATTAGCAGCCAGTGCAACTTTGACTTCTGCATTAAAAAGTCCGTGGAACTGTTTCACAACAACTTCATCAATCAGATTAATCAACTGCTGT

Y S F P L D Y K L S S G E Y F W V G Q K K P P Q P I V F D V N N E M I Q E F L L S T S N L L A Q V Y N I P P
2241 ATAGCTTCCCGCTGGATTATAAACTGAGCAGCGGTGAATATTTCTGGGTGGGCCAAAAAAAACCGCCTCAGCCGATTGTTTTTGACGTGAACAATGAAATGATCCAAGAATTTCTGCTGAGCACCAGCAATCTGCTGGCCCAGGTTTATAACATTCCTCC

C F D I N Y I I N V A K K I E V K P F E P K K V K I N M D E K N L N N I S I S F A E E E K I I D D F C K E
2401 GTGTTTCGATATTAACTATATCATCAACGTGGCGAAAAAAATCGAGGTGAAACCGTTTGAACCGAAAAAGGTGAAAATCAACATGGACGAGAAGAACCTGAATAACATTAGCATTAGCTTTGCCGAGGAAGAGAAGATCATTGACGATTTTTGTAAAGAG

L L N I P T N N I K I N P I E F D K D E Q T N L H V N F I Y A F S N L R A I N Y K I N T C D K L K A K I V
2561 CTGCTGAATATCCCGACCAACAACATTAAAATCAACCCGATCGAGTTCGATAAAGATGAACAGACCAATCTGCATGTGAACTTCATTTATGCCTTTAGTAATCTGCGTGCCATCAACTATAAGATCAATACCTGCGATAAACTGAAGGCCAAAATTGTTG

A G K I I P A L A T T T S I I T G L V G I E L L K Y V N Y Y D N I Q A Y V K L S D E Q R K K E K H D V L S Y
2721 CGGGTAAAATCATTCCGGCACTGGCAACCACCACCAGTATTATTACCGGTCTGGTGGGTATTGAACTGCTGAAATATGTGAACTACTACGACAACATTCAGGCCTATGTTAAACTGAGTGATGAGCAGCGCAAAAAAGAAAAGCATGATGTTCTGAGCTA

F K N A F I N S A L P L F L F S E P M P P L R M M D K E Y D E L M K G P V K A I P N G F S S W D K I V I S
2881 CTTCAAGAACGCCTTTATTAACAGCGCACTGCCGCTGTTTCTGTTCAGCGAACCGATGCCTCCGCTGCGTATGATGGATAAAGAATATGATGAACTGATGAAAGGTCCGGTTAAAGCAATTCCGAATGGTTTTAGCAGCTGGGATAAAATTGTGATCAGC

I K N G T I K D L I D H I N E K Y S I D V N L I S V G N A C L Y N C Y L P A H N K E R L N K P I H E L Y K
3041 ATCAAAAACGGCACCATCAAAGACCTGATTGATCACATCAATGAGAAGTATAGCATCGACGTGAATCTGATTAGCGTTGGTAATGCCTGTCTGTATAATTGTTATCTGCCTGCGCATAACAAAGAGCGTCTGAATAAACCGATTCACGAACTGTATAAGC

Q I S K Q D L L E D K N Y I I V E A S C S D Q D L V D V L I P S I Q F I Y K *
3201 AGATCAGTAAACAGGACCTGCTTGAGGATAAAAACTACATTATCGTTGAAGCCAGCTGTAGCGATCAGGATCTGGTGGATGTGCTGATTCCGAGCATCCAGTTTATCTACAAATGATAGTAACTCGAG
