# Supplementary material for: Ubiquitin activation is essential for schizont maturation in Plasmodium falciparum blood-stage development
Source: PLoS Pathog. 2020 Jun 22;16(6):e1008640. doi: 10.1371/journal.ppat.1008640 (PMC7332102; doi:10.1371/journal.ppat.1008640)
Supplement: S1 Table — (DOCX) [file ppat.1008640.s006.docx]

| **Gene name** | **Protein name** | **Number of ubiquitylation sites identified** | | | | | | | | | |
| --- | --- | --- | --- | --- | --- | --- | --- | --- | --- | --- | --- |
|  | **(PlasmoDB v.46)** | **All parasite stages** | | **Rings** | | **Trophozoites** | | **Schizonts** | | **Merozoites** | |
| PF3D7_0109000 | photosensitized INA-labeled protein PHIL1 (PHIL1) | 3 |  | |  | |  | | 3 | |  |
| PF3D7_0217500 | calcium-dependent protein kinase 1 (CDPK1) | 2 |  | |  | | 1 | | 2 | |  |
| PF3D7_0304100 | inner membrane complex protein 1e, putative (IMC1e) | 3 |  | |  | |  | | 3 | |  |
| PF3D7_0423500 | glideosome associated protein with multiple membrane spans 2 (GAPM2) | 2 |  | |  | |  | | 2 | |  |
| PF3D7_0515700 | glideosome-associated protein 40, putative (GAP40) | 2 |  | |  | |  | | 2 | |  |
| PF3D7_0522600 | inner membrane complex protein | 1 |  | |  | |  | | 1 | |  |
| PF3D7_0525800 | inner membrane complex protein 1g, putative (IMC1g) | 8 |  | |  | |  | | 8 | |  |
| PF3D7_0822900 | conserved Plasmodium protein, unknown function | 25 |  | |  | |  | | 25 | |  |
| PF3D7_1003600 | inner membrane complex protein 1c, putative (IMC1c) | 6 |  | |  | | 1 | | 6 | |  |
| PF3D7_1017500 | myosin essential light chain (ELC) | 5 |  | |  | |  | | 5 | |  |
| PF3D7_1222700 | glideosome-associated protein 45 (GAP45) | 4 |  | |  | |  | | 4 | |  |
| PF3D7_1246200 | actin I (ACT1) | 11 |  | |  | | 6 | | 11 | |  |
| PF3D7_1246400 | myosin A tail domain interacting protein (MTIP) | 3 |  | |  | |  | | 3 | |  |
| PF3D7_1251200 | coronin | 11 |  | |  | |  | | 11 | |  |
| PF3D7_1323700 | glideosome associated protein with multiple membrane spans 1 (GAPM1) | 2 |  | | 1 | | 1 | | 2 | |  |
| PF3D7_1342600 | myosin A (MyoA) | 6 |  | |  | |  | | 6 | |  |
| PF3D7_1351700 | inner membrane complex protein 1f, putative (IMC1f) | 8 |  | |  | |  | | 8 | |  |
| PF3D7_1460600 | inner membrane complex sub-compartment protein 3 (ISP3) | 1 |  | |  | |  | | 1 | |  |
