# Supplementary material for: Ubiquitin activation is essential for schizont maturation in Plasmodium falciparum blood-stage development
Source: PLoS Pathog. 2020 Jun 22;16(6):e1008640. doi: 10.1371/journal.ppat.1008640 (PMC7332102; doi:10.1371/journal.ppat.1008640)
Supplement: S2 Table — (DOCX) [file ppat.1008640.s007.docx]

| **Gene name** | **Protein name** | **Number of ubiquitylation sites identified** | | | | | | | | | |  |
| --- | --- | --- | --- | --- | --- | --- | --- | --- | --- | --- | --- | --- |
|  | **(PlasmoDB v.46)** | **All parasite stages** | | **Rings** | | **Trophozoites** | | **Schizonts** | | **Merozoites** | |  |
| PF3D7_0610400/  PF3D7_0617900 | histone H3 (H3) | | 5 | | 1 | | 3 | | 2 | | 5 | |
| PF3D7_0617800 | histone H2A (H2A) | | 5 | | 1 | | 4 | | 3 | | 5 | |
| PF3D7_0714000 | histone H2B variant (H2B.Z) | | 5 | | 1 | | 2 | | 3 | | 5 | |
| PF3D7_0320900 | histone H2A variant (H2A.Z) | | 4 | |  | |  | | 2 | | 4 | |
| PF3D7_1105000 | histone H4 (H4) | | 4 | | 1 | | 1 | | 1 | | 4 | |
| PF3D7_1105100 | histone H2B (H2B) | | 4 | | 2 | | 4 | | 4 | | 4 | |
| PF3D7_0617900 | histone H3 variant (H3.3) | | 5 | | 1 | | 1 | | 1 | | 5 | |
