# Supplementary material for: Ubiquitin activation is essential for schizont maturation in Plasmodium falciparum blood-stage development
Source: PLoS Pathog. 2020 Jun 22;16(6):e1008640. doi: 10.1371/journal.ppat.1008640 (PMC7332102; doi:10.1371/journal.ppat.1008640)
Supplement: S3 Table — (DOCX) [file ppat.1008640.s008.docx]

| Primer | Sequence |  |
| --- | --- | --- |
| 107for | ATTGTGATCGATTAAATCTTTAA | Guide 107 |
| 107rev | AAACTTAAAGATTTAATCGATCA |  |
| 109for | ATTGTTATTTGCCTGCACACAACA | Guide 109 |
| 109rev | AAACTGTTGTGTGCAGGCAAATAA |  |
| 111for | ATTGTTAAGACATCTACGAGGTCT | Guide 111 |
| 111rev | AAACAGACCTCGTAGATGTCTTAA |  |
| 113 | gatcCCGCGGTGATGAGTGCACAAATATATATATATATATAT |  |
| 114 | gatcAAGCTTCAAAACGGTGATGGGTATATATACTTCCTTGTT |  |
| 116 | GTTCATATAAACATCAAAATGAATCAACAATGATTC |  |
| 121 | ATATTTCCATATCCTTTGCCGAAGAAGAAAAAA | screening primers |
| GFPrev | CTCCAGTGAAAAGTTCTTCTCC |  |
| HArev | CCTTTACCGCGGTCAAGCGTAAT |  |
